# Discrepancies in the efficacy of H5 inactivated avian influenza vaccines in specific-pathogen-free chickens against challenge with the Egyptian H5N8 clade 2.3.4.4 Group B virus isolated in 2018

**DOI:** 10.14202/vetworld.2021.2131-2141

**Published:** 2021-08-20

**Authors:** Amena Abd El-Moeid, Ayman Hany EL-Deeb, Marwa Fathy Elsaied, Reem Ahamed Soliman, Mounir Mohamed EL-Safty, Hussein Aly Hussein

**Affiliations:** 1 Department of Virology, Faculty of Veterinary Medicine, Cairo University, Giza, Egypt; 2 Central Laboratory for Evaluation of Veterinary Biologics, Abassia, Cairo, Egypt

**Keywords:** avian influenza, avian influenza viruses H5N8, clade 2.3.4.4, HPAI, H5 vaccines

## Abstract

**Background and Aim:**

Highly pathogenic avian influenza H5N8 virus of clade 2.3.4.4 was newly emerged to Egypt and firstly detected in carcasses of wild birds in November 2016. This study assessed the protection efficacy and virus shedding reduction of three different inactivated avian influenza (AI) H5 (H5N1, H5N2, and H5N3) commercial vaccines against challenge with two newly emerging highly pathogenic AI virus H5N8 Egyptian isolates in specific-pathogen-free (SPF) chicks.

**Materials and Methods:**

10-day-old SPF chicks (n=260) were divided into 20 groups (n=13). Groups 1-5 were vaccinated through the subcutaneous route (S/C) with 0.5 mL of H5N1 vaccine, Groups 6-10 were vaccinated (S/C) with 0.5 mL of H5N2 vaccine, and Groups 11-15 were vaccinated (S/C) with 0.5 mL of H5N3 vaccine. Positive control groups (16-19) were challenged at 25 and 31 days old (2 and 3 weeks post-vaccination [PV]) using H5N8 clade 2.3.4.4 A/duck/Egypt/F13666A/2017(H5N8) and H5N8 clade 2.3.4.4 A/chicken/Egypt/18FL6/2018(H5N8). Group 20 was left non-vaccinated as a control. All vaccinated groups were divided and challenged with both viruses at 25 and 31 days of age. The viral challenge dose was 0.1 mL of 10^6^ EID_50_/0.1 mL titer/chick, and it was administered oronasally. All chicks were kept in isolators for 14 days after each challenge. Sera samples were collected weekly and at 2 weeks post-challenge (PC) to detect a humoral immune response. PC mortalities were recorded daily for 10 days to calculate the protection percentages. Tracheal swabs were collected from the challenged chicks in different groups at 3, 5, 7, and 10 days PC. Kidneys and spleens were collected at 3, 5, 7, and 10 days PC and kept in formalin for histopathological examination to assess lesions and severity scores. Tracheal swabs were inoculated in 10-day-old SPF embryonated chicken eggs for virus titration and to calculate shedding levels.

**Results:**

All studied vaccines displayed 70-100% protection within 10 days PC. Hemagglutination inhibition results from sera samples revealed antibody titers ranging from 0.6 to 5.4 log_2_ starting at 1-week PV with the highest titers at 4 weeks PV. Challenged SPF chickens exhibited a notable reduction in virus shedding, with an average of 1.5-2 log_10_, compared to control birds. Various histopathological lesions with different scores were detected.

**Conclusion:**

Our findings suggest that the inadequate virus shedding reduction and protection efficacy of studied vaccines were variable and that the type of vaccine to be used under field conditions should be reconsidered. Study of the variability between the Egyptian old emerged AI (AIV) 2017 H5N8 strains and the new emerging AIV 2018 H5N8 is required to achieve optimal protection and limit the current economic losses.

## Introduction

Highly pathogenic avian influenza virus (HPAIV) H5N8 strains were first detected in 2010 in types of wild birds in Asia, and the virus subsequently spreads to different areas worldwide [1]. In Egypt, the virus was first reported at the end of 2016 [1]. Egypt is located at a crossing point between Europe, Africa, and Asia which increases the risk of the different viruses of avian influenza being spread by migrating wild birds as the overlapping migrations of such birds represent the first line to spread the HPAIV H5N8 strains. In Egypt, these viruses were first detected in November 2016 in the carcasses of wild migratory birds [1], specifically the common coot, which was included in targeted surveillance for AIVs in migratory birds conducted by the community Animal Health Outreach. These H5N8 strains were classified as HPAIV due to the presence of multibasic cleavage sites of the HA that characterizes the high pathogenicity of AIVs in the currently characterized H5N8 viruses in the motif PLREKRRKR#GLF (# denotes cleavage site) [2]. The virus was also detected in another wild bird (green-winged teal). New isolates were recorded in unvaccinated domestic chickens and flocks of ducks in 2017 and 2018 [3-5]. New strains of HPAI H5N8 are detected yearly and have caused economic losses in the Egyptian poultry sector [6]. The controlling of AI infections in Egypt relies primarily on a routine vaccination strategy, which plays a key role as a preventive tool designed to minimize losses in poultry production by limiting the spread of infection, depending on the vaccine’s ability to reduce morbidity, thereby restricting the spread of AI viruses [7,8]. Controlling virus shedding from infected animals reduces both the likelihood of more circulating viruses being generated and the risk of more newly reassorted strains being produced. Besides decreasing the mortality [9], however, the wide range of variation of the HA of the AIVs, coupled with antigenic variation within the same subtype, leads to failure of the previous routine strategy against those newly emerging H5N8 strains and consequently mortalities and production losses even in vaccinated flocks [6].

Many commercial different AI vaccines are employed in combating H5 Infections in Egypt [10]. However, the uninterrupted mortalities in vaccinated flocks from H5N8 infection have raised the question about the variation between the previously detected Egyptian HPAIV 2017 H5N8 strains and the newly emerging HPAIV 2018 H5N8 strains in respect of their reactivity to the currently used commercial H5 vaccines. Routine evaluation of the most commonly used commercial AI vaccines against the newly emerged H5N8 strains with different pathogenicity and clinical presentation of AI (unpublished data) is required urgently to develop the most appropriate vaccination strategy.

In this study, we designed an experiment to assess some of the widely used commercial AI inactivated vaccines (H5N1, H5N2, and H5N3) when employed to immunize against challenge with two different Egyptian HPAI H5N8 isolates clade 2.3.4.4 at 2 and 3 weeks post-vaccination (PV) in specific-pathogen-free (SPF) chickens in isolators. We recorded protection rates, antibody titers, virus shedding titers, and the histopathological changes in kidney and spleen.

## Materials and Methods

### Ethical approval

This study was approved by the Institutional Animal Care and Use Committee at Central Laboratory for Evaluation of Veterinary Biologics (CLEVB), Cairo, Giza, Egypt.

### Study period and location

The study was conducted in November 2018. The samples were processed at CLEVB, Cairo, Egypt, Department of Virology, Faculty of Veterinary Medicine, Cairo University, Giza, Egypt and Animal Health Research Institute, Dokki, Giza.

### Viruses

Two different HPAI H5N8 viruses were isolated in 2017 and 2018. The 2017 isolate was of duck origin: (clade 2.3.4.4 A/duck/Egypt/F13666A/2017(H5N8)) with GenBank accession No. (MH498622.1). The 2018 isolate was of chicken origin [clade 2.3.4.4 A/chicken/Egypt/18FL6/2018(H5N8)] with GenBank accession No. (MH986133.1). We used these two viruses in challenge experiments at a titer of 10^6^ EID50/0.1 mL.

### Vaccines

We used three different commercial inactivated vaccines (H5N1, H5N2, and H5N3) in current use to assess their efficacy and investigate the reduction in shedding achieved in vaccinated challenged chicks (Table-1).

**Table-1:** List of H5 inactivated commercial vaccines used for the immunization of SPF poultry against H5N8.

Vaccine name	Virus used	Lineage	Manufacturing country	HA nucleotide sequence % similarity to Egyptian 2017 H5N8 used in challenge	HA nucleotide sequence % similarity to Egyptian 2018 H5N8 used in challenge
H5N1	RG A/duck/Anhui/1/2006(H5N1) (Re-5)	Clade 2.3.4	China	94	91.7
H5N2	A/chicken/Mexico/232/1994 (H5N2)	Classic	Mexico	80	75.8
H5N3	A/chicken/Vietnam/C58/2004 (H5N3)	Clade 1	USA	91	90

### SPF chicks and eggs

We obtained two hundred and sixty 1-day-old SPF chicks from Koum Oshiem SPF Chicken Farm, Fayoum, Egypt, and kept them in biosafety HEPA filtered isolators, with food and water supplied *ad libitum*. For virus titration, we used SPF embryonated chicken eggs obtained from Koum Oshiem SPF Chicken Farm, Fayoum, Egypt.

### Vaccination and challenge trials

#### Vaccination

We divided the 260 SPF chicks into 20 groups (n=13). All chicks were raised in isolators. At 10 days’ old, Groups 1-5 were vaccinated through the subcutaneous route (S/C) with 0.5 mL of H5N1 vaccine, Groups 6-10 were vaccinated S/C with 0.5 mL of H5N2 vaccine, and Groups 11-15 were vaccinated S/C with 0.5 mL of H5N3 vaccine as per the manufacturers’ recommendations. We inoculated Groups 16-19 with an equal dose of phosphate-buffered saline and used them as positive controls. We left Group 20 non-vaccinated as a control (Table-2).

**Table-2:** Challenge trials.

Groups	Vaccination at 10 days old	Challenge	Age/day	Weeks PV
H5N1-vaccinated groups:			
Group 1	H5N1	H5N8 2017	25	2
Group 2	H5N1	H5N8 2018	25	2
Group 3	H5N1	H5N8 2017	31	3
Group 4	H5N1	H5N8 2018	31	3
Group 5	H5N1	Non	-	-
H5N2-vaccinated groups:			
Group 6	H5N2	H5N8 2017	25	2
Group 7	H5N2	H5N8 2018	25	2
Group 8	H5N2	H5N8 2017	31	3
Group 9	H5N2	H5N8 2018	31	3
Group 10	H5N2	Non	-	-
H5N3-vaccinated groups:			
Group 11	H5N3	H5N8 2017	25	2
Group 12	H5N3	H5N8 2018	25	2
Group 13	H5N3	H5N8 2017	31	3
Group 14	H5N3	H5N8 2018	31	3
Group 15	H5N3	Non	-	-
Control positive groups:			
Group 16	-	H5N8 2017	25	2
Group 17	-	H5N8 2018	25	2
Group 18	-	H5N8 2017	31	3
Group 19	-	H5N8 2018	31	3
Control negative group:			
Group 20	-	-	-	-

H5N1=AI inactivated H5N1 vaccine, H5N2=AI inactivated H5N2 vaccine, H5N3=AI inactivated H5N3 vaccine, H5N8 2017=(clade 2.3.4.4 A/duck/Egypt/F13666A/2017(H5N8)), H5N8 2018=(clade 2.3.4.4 A/chicken/Egypt/18FL6/2018(H5N8))

#### Challenge trials

We divided the groups of SPF chicks, as shown in Table-2. All challenged birds were inoculated oronasally with 0.1 mL of the challenge virus. The dose was determined based on the standard dose used in Egypt to evaluate all HPAI-H5 vaccines submitted to the CLEVB [10]. After administering the challenge, we monitored the birds daily for 10 days. We recorded clinical signs, morbidity, and mortality post-challenge (PC). All experiments in which we used HPAI viruses in work with animals were reviewed by the institutional biosecurity committee and were conducted in biosecurity level-3-enhanced (BSL3E) facilities at the CLEVB.

### Serology and antibody assay

We collected sera samples before vaccination to confirm that the SPF chickens were free from any infectious diseases, such as AI infections. We determined antibody titers in sera samples collected weekly PV in vaccinated control groups and after 2 weeks PC by a hemagglutination inhibition (HI) test using the universal reference H5 antigen (GD-Netherland) as a heterologous antigen in addition to two local homologous antigens prepared from the two challenge viruses. The HI assay was carried out according to the protocol of the World Organization for Animal Health (OIE) [11].

### Determination of virus shedding

We collected tracheal swabs at 3, 5, 7, and 10 days PC to determine virus shedding for all challenged groups. We performed the procedures according to the laboratory manual for the isolation, identification, and characterization of avian pathogens [12]. We calculated the virus shedding titers following the method of Spearman–Karber [12]. We reported the results as EID_50_/0.1 mL equivalents, with the lower limit of detection being <1 log10 EID_50_/0.1 mL [6].

### Histopathological examination

To determine the histopathological changes caused by the challenge, we collected spleens and kidneys from each group at 3, 5, 7, and 10 days PC, fixed them in 10% formol saline embedded in paraffin, cut them into sections 4 μm thick, and stained the sections with hematoxylin and eosin [13]. We recorded and calculated pathological scores as previously reported by Gibson-Corley *et al*. [14] for chicken groups challenged against A/chicken/Egypt/18FL6/2018(H5N8) virus due to variability in protection and shedding in those groups.

### Statistical analysis

We carried out multiple comparisons using two-way analysis of variance. We used the least standard difference to both analyze the differences in means and standard error between HI titers and to compare the results of virus shedding titers from the tracheal swabs for the various groups. All statistical tests were performed using p<0.05. All graphs and survival curves were created using Prism 7 (GraphPad Co., San Diego, USA).

## Results

### Protective percentages in vaccinated and challenged chicks

**Figure-1:**
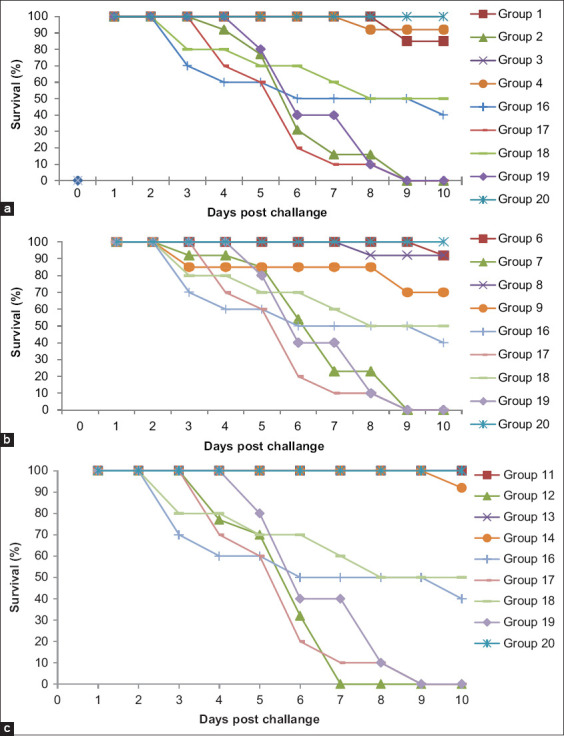
Survival curves of unimmunized and immunized birds using three different H5 inactivated vaccines post-challenge with 2 HPAI H5N8 isolates (2017 and 2018) at two different ages (25 and 31 day old). (a) H5N1-vaccinated groups (b) H5N2-vaccinated groups (c) H5N3-vaccinated groups.

When challenged with 2017 and 2018 H5N8 isolates, the H5N1-vaccinated groups (G1–G4) displayed 85% and 0% protection, respectively, when challenged at two WPV (weeks PV) and 100% and 92% protection, respectively, when challenged at three WPV (Figure-1a). When challenged with 2017 and 2018 H5N8 isolates, the H5N2-vaccinated groups (G6–G9) displayed 92% and 0% protection, respectively, when challenged at two WPV and 92% and 70% protection when challenged at three WPV (Figure-1b). When challenged with 2017 and 2018 H5N8 isolates, the H5N3-vaccinated groups (G11–G14) displayed 100% and 0% protection, respectively, when challenged at two WPV and 100% and 92% protection, respectively, when challenged at three WPV (Figure-1c). There was no evidence of either morbidity or mortality in Groups 5, 10, 15, and 20. When challenged with 2017 and 2018 H5N8 isolates, positive controls (G16–G19) displayed 40% and 0% protection, respectively, when challenged at two WPV and 50% and 0% protection, respectively, when challenged at three WPV (Figure-1 and Table-3).

**Table-3:** The protection percentages of challenged groups.

Vaccine	Challenge time	H5N8 challenge isolate	

		H5N8 2017	H5N8 2018
H5N1	2 WPV	85%	0%
	3 WPV	100%	92%
H5N2	2 WPV	92%	0%
	3 WPV	92%	70%
H5N3	2 WPV	100%	0%
	3 WPV	100%	92%
-	2 WPV	40%	0%
-	3 WPV	50%	0%

H5N1=AI inactivated H5N1 vaccine, H5N2=AI inactivated H5N2 vaccine, H5N3=AI inactivated H5N3 vaccine, H5N8 2017=(clade 2.3.4.4 A/duck/Egypt/F13666A/2017(H5N8)), H5N8 2018=(clade 2.3.4.4 A/chicken/Egypt/18FL6/2018(H5N8))

#### Humoral response to vaccination and challenge

**Figure-2:**
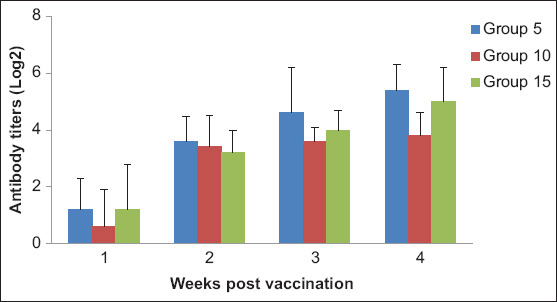
Column charts showing serum hemagglutination inhibition titers of H5 avian influenza virus post-vaccination (expressed as log_2_). Group 5: H5N1 vaccinated group, Group 10: H5N2 vaccinated group and Group 15: H5N3 vaccinated group.

**Figure-3:**
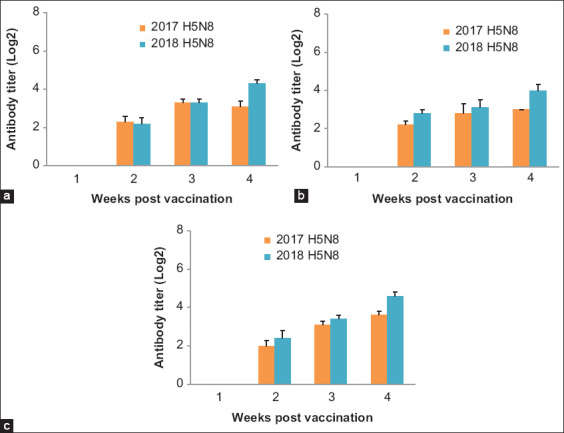
Column charts showing mean serum hemagglutination inhibition titers of H5 avian influenza virus (expressed as log_2_) in vaccinated groups with three different H5 inactivated vaccines using challenge viruses H5N8 (2017 and 2018) as antigens. (a) Group 5: H5N1 vaccine (b) Group 10: H5N2 vaccine (c) Group 15: H5N3 vaccine.

The collected pre-vaccination samples were free from any antibodies against AI infections (H5N1, H9N2, and H5N8). In vaccinated non-challenged groups, HI titers using the universal reference H5 antigen (GD-Netherland) revealed a significant difference at four WPV between the H5N1-vaccinated group and the H5N2-vaccinated group, with the highest titer being in the H5N1 group (5.4 log_2_), followed by the H5N3 group (5 log_2_), compared to the H5N2 group (only 3.8 log2) (p<0.05) (Figure-2). Conversely, when using homologous antigens prepared from the challenge viruses (HPAI H5N8, clade 2.3.4.4: 2017 and 2018), we found differences in the HI titers obtained. HI titers obtained against the 2017 and 2018 antigens differed significantly at four WPV between the H5N1-vaccinated group (3.1-4.3 log_2_) and the H5N3-vaccinated group (3.6-4.6 log_2_) (Figure-3) with observing the presence of lower individual antibodies values as follows: At two WPV, the H5N3-vaccinated group displayed 20% of 1 log_2_ against the 2017 H5N8 antigen, whereas both the H5N1-vaccinated and the H5N3-vaccinated groups displayed 20% of 1 log_2_ against the 2018 H5N8 antigen, compared to 0% of 1 log_2_ in all groups using the universal antigen (Table-4).

**Table-4:** Mean HI antibody titers percentages in vaccinated birds at 2 weeks post-vaccination.

Vaccine type	Mean HI titers (Log_2_) percentage			

	Used antigen	1	2	3
H5N1	2017	0	50	50
	2018	20	40	40
H5N2	2017	0	80	20
	2018	0	20	80
H5N3	2017	20	60	20
	2018	20	20	60

H5N1=AI inactivated H5N1 vaccine, H5N2=AI inactivated H5N2 vaccine, H5N3=AI inactivated H5N3 vaccine, H5N8 2017=(clade 2.3.4.4 A/duck/Egypt/F13666A/2017(H5N8)), H5N8 2018=(clade 2.3.4.4 A/chicken/Egypt/18FL6/2018(H5N8))

At three WPV, the H5N2-vaccinated group displayed 2 log_2_ HI titers in 66% and 16% against the H5N8 2017 and H5N8 2018 antigens, respectively, and only 14% in the H5N3-vaccinated group against the 2017 H5N8 antigen compared to 0% of 2 log_2_ in all groups using the universal antigen (Table-5).

**Table-5:** Mean HI antibody titers percentages in vaccinated birds at 3 and 4 weeks post-vaccination.

WPV	Vaccine type	Mean HI titers (Log_2_) percentage (%)				

		Used antigen	2	3	4	5
3	H5N1	2017	0	66	34	0
		2018	0	66	34	0
	H5N2	2017	66	0	17	17
		2018	16	67	0	16
	H5N3	2017	14	57	29	0
		2018	0	60	40	0
4	H5N1	2017	16	52	33	0
		2018	0	0	66	34
	H5N2	2017	0	100	0	0
		2018	0	33	34	33
	H5N3	2017	0	33	67	0
		2018	0	0	33	67

H5N1=AI inactivated H5N1 vaccine, H5N2=AI inactivated H5N2 vaccine, H5N3=AI inactivated H5N3 vaccine, H5N8 2017=(clade 2.3.4.4 A/duck/Egypt/F13666A/2017(H5N8)), H5N8 2018=(clade 2.3.4.4 A/chicken/Egypt/18FL6/2018(H5N8))

At four WPV, only 16% of the H5N1-vaccinated group against 2017 H5N8 antigen displayed HI titers of 2 log_2_, compared to 0% of 2 log_2_ in all groups using the universal antigen. In addition, we found 3 log_2_ titers at 33% in the H5N3- and H5N2-vaccinated groups against the 2017 and 2018 antigens and 52% in the H5N1-vaccinated group against the 2017 antigen, with 100% in the H5N2-vaccinated group against the 2017 antigen (as shown in Table-5) compared to 0% of 3 log_2_ in all groups using the universal antigen, apart from the H5N2-vaccinated group, which displayed 28% of 3 log_2_ against the universal antigen.

**Figure-4:**
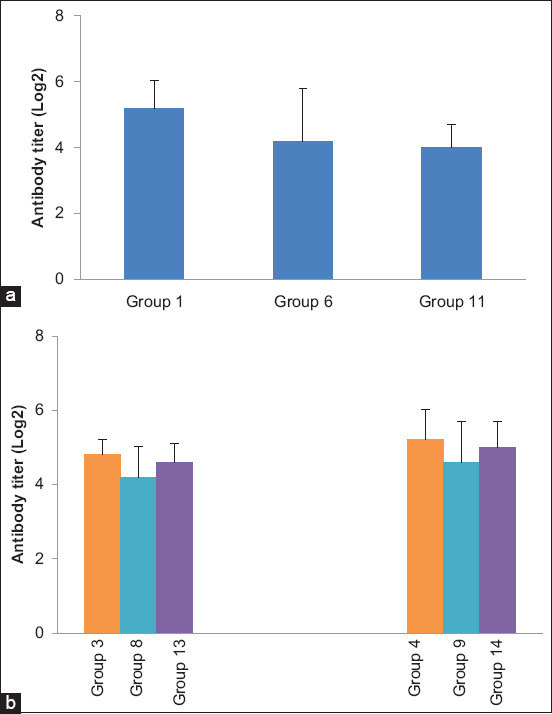
Column charts showing mean serum hemagglutination inhibition titers of H5 avian influenza virus (expressed as log_2_) post-challenge. (a) 2 WPV challenge. (b) 3 WPV challenge. WPV=Weeks post-vaccination.

Conversely, we did not observe any significant difference in HI titers PC in all vaccinated two WPV (25 days old) or three WPV (31 days old) challenged groups, irrespective of the vaccine type (Figure-4).

### Virus shedding after challenge with HPAI H5N8 clade 2.3.4.4 2017 and 2018 Egyptian isolates

**Figure-5:**
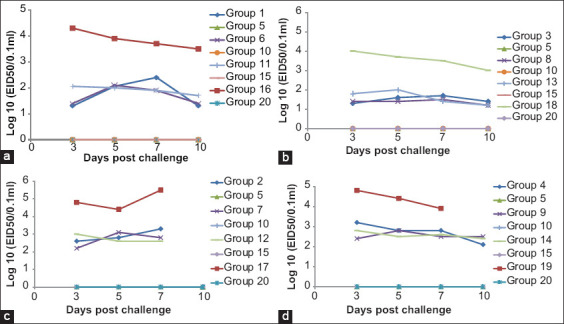
Line charts showing virus titration in ECE from tracheal swabs (expressed as log 10) in different groups and different time conditions. (a) Challenge using 2017 H5N8 isolate at 2 weeks post-vaccination (PV). (b) Challenge using 2017 H5N8 isolate at 3 weeks PV. (c) Challenge using 2018 H5N8 isolate at 2 weeks PV. (d) Challenge using 2018 H5N8 isolate at 3 weeks PV.

With the H5N8 2017 challenge virus, we found a significant difference in shedding titers between the H5N1- and H5N2-vaccinated groups versus the H5N3-vaccinated group on the 3^rd^ day PC, with the highest shedding titer in the H5N3 group (2.06 log_10_), and also between the H5N1- and H5N3-vaccinated groups on the 10^th^ day PC when challenged at two WPV (25 days old) (Figure-5a), whereas in those challenged at three WPV (31 days old), we found no significant difference at different days PC in all groups (Figure-5b). With the H5N8 2018 challenge virus, we found a significant difference in shedding titers between all vaccinated groups at all days PC when challenged at two WPV (Figure-5c) and on the 3^rd^ day PC when challenged at three WPV. At the 10^th^ day PC in those challenged at three WPV, we found a significant difference between the H5N1-vaccinated groups versus the H5N2- and H5N3-vaccinated groups (Figure-5d). Positive controls (G16 to G19) displayed an average of 1.1-3 log10 shedding titers higher than the vaccinated challenged groups (Figure-5).

### Histopathological examination of organs:

Only tissues collected from the vaccinated and challenged with 2018 H5N8 virus groups were examined for histopathological changes compared to the positive and negative controls. In Group 2, the kidneys displayed severely congested blood vessels, degenerated tubules with contracted glomeruli, interstitial hemorrhage, and marked vacuolar degeneration of renal tubules associated with few hyaline casts, and the spleens displayed mild depletion of lymphocytes and mild congestion (Table-6). In Group 7, the kidneys displayed pronounced congested blood vessels with degenerated tubules, focal mononuclear cell infiltration, multiple tubular cysts, contracted glomeruli, and thickening of the blood vessel walls with hyperplasia. The spleens displayed mild depletion and hemorrhage and mild hemorrhage and focal thickening of the splenic capsule (Table-6). In Group 12, the kidneys displayed mild congested blood vessels with denudation, degeneration of the renal tubules, and vacuolation of mesangial cells characterized by thrombus formation. The spleens displayed mild focal depletion of lymphocytes accompanied by thickening of the blood vessel walls (Table-6). In Group 17, the kidneys displayed interstitial congestion, mononuclear cell infiltration with thrombus formation, degenerated tubules with cyst formation, and contracted glomeruli, and the spleen displayed proliferation of sheathed capillaries and depletion of the lymphocytes (Table-6).

**Table-6:** The score of pathological lesions for all challenged groups against 2018 H5N8 virus.

Days post-challenge	Group	Organs		Group	Organs	
	
		Kidney Spleen			Kidney Spleen	
	
		2 WPV challenge			3 WPV challenge	
3	2	+++	+	4	++	+
5		+++	+		+	+
7		+++	+		++	+
10	NS				++	+
3	7	++	+	9	+++	−
5		+++	+		++	+
7		++	+		++	+
10	NS				++	+
3	12	+	+	14	+	+
5		+	+		+	+
7		+	+		++	+
10	NS				++	+
Pool	17	+++	+	19	+++	+
Pool	5	−	−	5	−	−
Pool	10	−	−	10	−	−
Pool	15	−	−	15	−	−
Pool	20	−	−	20	−	−

WPV=Weeks post-vaccination, (−): No lesions/(+): Mild/(++): Moderate/(+++): Severe, NS=Non-survival

In Groups 4 and 9, the kidneys showed the same lesions as mild intertubular congestion with degeneration, denudation of the renal tubules, and thickening of the wall of the ureter. Only Group 9 showed interstitial focal mononuclear cell infiltration with thrombus formation (Table-6). In Group 4, the spleens displayed mild depletion, with mildly congested blood vessels and mild focal edema, whereas those of Group 9 appeared to range from normal to showing mild depletion of lymphocytes and congested blood vessels with endotheliosis (Table-6). In Group 14, the kidneys displayed exhibited congested blood vessels with perivascular mononuclear cell infiltration, thickening of the wall of the ureter, and focal interstitial edema with extravasated red blood cells, whereas the spleens showed mild depletion and proliferation of sheathed capillaries associated with mild hemorrhage.

The organs of Groups 5, 10, 15, and 20 showed normal histological characters without any changes (Table-6), whereas the kidneys of Group 19 displayed interstitial congestion, mononuclear cell infiltration, thrombus formation, degenerated tubules with cyst formation, and contracted glomeruli, and the spleens exhibited proliferation of sheathed capillaries and depletion of the lymphocytes (Table-6).

## Discussion

Poultry production in Egypt has suffered economic losses since the emergence of HPAIV H5N8 strains [10] clade 2.3.4.4b in 2016 [1], with new strains being reported continuously in 2017 [3,4] and 2018 [5]. In this study, we have compared the efficacy of three different AI H5-inactivated vaccines (H5N1, H5N2, and H5N3) that are commonly used in the field against two HPAI H5N8 viruses (2017 and 2018 official isolates used for the evaluation of commercial vaccines) that differ in their pathogenicity and clinical presentation of disease in both SPF and commercial birds (unpublished data). Groups of vaccinated birds were challenged at two and three WPV. Our findings have shown the ability of H5N1 and H5N3 vaccines to act efficiently against both challenge viruses (2017 and 2018 H5N8) after three WPV, with both vaccines inducing protection that ranged from 92% to 100%. In contrast, the H5N2 vaccine did not protect against the 2018 H5N8 isolate at three WPV, with the level of protection being just 70%. Conversely, all tested vaccines could not provide any level of protection against the 2018 challenge H5N8 isolate at two WPV (0%). The level of protection recommended by the OIE is not <80% [11]. Protection studies revealed differences in the challenged groups, with the tested H5N3 vaccine showing 100% protection against the 2017 H5N8 AI isolate when challenged at two WPV. The three different vaccines used in this study exhibited the ability to protect the birds from lethal disease after being challenged with the 2017 H5N8 isolate, which indicates that the vaccines used are successful against challenge with this isolate as early as two WPV, whereas in birds challenged with the 2018 H5N8 isolate, they showed 0% protection within just 6-8 days PC, and the birds displayed typical signs of AI and patterns similar to those seen in the control unvaccinated challenged groups (mortality reaching 100%). Similar levels of mortality with H5N8 have been observed recently by others [15] and in another challenge trial using 2018 and 2019 Egyptian isolates of chicken origin [7,16]. It has been reported previously that the absence of early induction of antibodies in vaccinated birds may be a contributory cause of the lack of early protection when these birds were challenged [10]. Of course, there are many explanations of such variation in protection against both challenge viruses. First, it could be attributed to the diversity, both antigenic and genetic, of the 2018 H5N8 isolate compared to the 2017 isolate [16]. A second explanation is the time of introduction of these viruses from migratory birds to chickens and of their detection in relation to season and species spillover [3]. Third, there is a gap before relatively high cross antibody titers are detected, and this can be from two to four WPV [10]. By three WPV, only the H5N2 vaccine failed to protect against the 2018 H5N8 isolate (70% protection).

Obviously, the differences observed in the clinical presentations, infection rates, and mortality patterns between the vaccinated and unvaccinated challenged groups were primarily due to the difference in both challenge viruses, as underpinned by similar findings in control unvaccinated challenged chickens with the H5N8 AI virus [16,17]. The AI H5N8 viruses were introduced in two different seasons and reported each year as a new introduction [3-5]. Genetic and antigenic variations have been confirmed in these strains in Egypt.

Such variability reflects on both antigenicity and pathogenicity. An earlier study found that the enormous use of various H5N1 and H5N2 vaccines pits vaccination pressure on the virus, causing selection of escape mutations at antigenic sites and leading to vaccination failure [18,19].

Although both the H5N8 isolates in this study were HPAIVs, the mortality from the 2017 isolate did not exceed 60%, which is similar to the initial mortality percentage seen in flocks of chicken and ducks, with the first detection of these viruses associated with mortality rates of up to 70% in farm flocks [3]. Another study [4] has reported mortalities ranging from 29% to 52% in unvaccinated flocks. In the current study, mortalities from 2018 isolates reached 100% by the 8^th^ day PC, with very severe clinical signs in positive control groups. Although the previous reports have indicated that HPAI H5N8 viruses exhibit asymptomatic disease in geese and ducks with prolonged virus shedding [20], the increased adaptation of the virus in chickens may be the main cause of such changes observed within these 2.3.4.4 clade HPAIVs [21,22], as continuous adaptation and enhanced replication through point mutations for H5N8, as one of the AIVs, have been reported [23]. Similarly, clade 2.3.4.4b H5N8 viruses have diversified into five genotypes (Gt1–Gt5) through constant evolution and reassortment [16,24]. Furthermore, these observed typical AI signs with H5N8 in chickens that are like those reported for the H5N1 strains, including cyanotic combs and wattles, edema of the head, hemorrhaging of the shank of the leg, and a 100% mortality rate, which indicated the vigorous evolution and host adaptation of those viruses [10].

Our serological results revealed the presence of considerable levels of antibodies, proving that the vaccines successfully induced an immune response. However, this is not indicative of the typical protection from mortality; chicken groups did not show the level recommended by OIE, which has determined that the minimum HI serological titers in birds to protect from mortality should be 1/32 [11]. Hence, we do not consider those measured antibodies to be the only factor contributing to protection. At three WPV, the H5N2-vaccinated groups challenged with 2018 H5N8 exhibited HI titers (just 4.6 log_2_) that were lower than the recommended level, and this was typically reflected in an unacceptable level of protection against mortality (70%). This may be due to the presence of variations in their antigenic sites [25] in relation to the challenge H5N8 viruses (differences in sequences homology between the vaccine seed virus and the challenge H5N8 viruses). This could reduce the reactivity of the same vaccines with the change of the virus challenge isolate, which is in line with the study of Swayne *et al*. [26], who found that the closer the similarity of the HA gene sequence between the vaccine and field viruses, the greater the protection conferred and the greater the reduction in replication of the challenge virus in the respiratory tract. Moreover, the changes in the pathogenicity of the virus led to reduced protection, although the level of HI titers remained unchanged; however, further studies are required for confirmation and detection of such variations. Recent studies have reported such variation between the H5 vaccines and the circulating H5N8 viruses and confirmed its impact on the protection conferred and the antibodies induced by the vaccines [6,10]. In the current study, these differences were also detected in the non-specific antibodies that appeared in titers at different weeks PV (1 log_2_ at two WPV and 2 log_2_ at three and four WPV) when we tested sera samples for HI titers using the two challenge viruses as antigens. Differences in HI titers between vaccinated birds in different groups may be due to differences in the antigens used in HI testing, as confirmed using the homologs and heterologous antigens in the current study [7,10].

Typically, the main function of any appropriate vaccine is to reduce mortality and, to some extent, virus shedding; this requires antigenic matching between the immunized vaccine and the challenge virus strain [27] to avoid production of escape mutants. There have been many studies aimed at evaluating commercial and experimental H5 vaccines against newly emerged HPAIV infections, and these have had variable results [6,10,17,28]. However, there has been concern about the failure of many vaccines to inhibit virus shedding [28]. In the current study, all vaccinated challenged groups displayed partial virus shedding, which, in accordance with a previous study in 2017 [6], we attributed to the genetic dissimilarity and poor reactivity between the H5 commercial vaccines used here and the H5N8 viruses currently in circulation. These findings are supported by the OIE recommendation [11] that the minimum HI serological titers in field birds should be >1/128 to provide a reduction in replication and shedding of the challenge virus. None of the groups in our study reached this level. A direct correlation between a high degree of virus shedding and the transmissibility of the virus among chickens has been confirmed [16]. In addition, the reassortant AIVs are thought to have developed and adapted from and gained dominance over previously circulating genotypes found in the Egyptian poultry flocks [29] by means of unknown selective gain.

HPAIVs have multiple basic amino acids (arginine and lysine) at their HA0 cleavage sites [30,31,32] and they appear to be capable of being cleaved by ubiquitous proteases. These viruses can replicate and spread throughout the host bird, damaging vital organs and tissues, and thus causing disease and death [33]. In the current study, this was clearly seen in histopathological lesions. Trypsin-like proteases are essential for activation of cleavage of the HA and so play a key role in viral pathogenicity [34,35]. In our study, the kidneys of birds challenged at early ages displayed more prominent histopathological lesions than did the kidneys of birds challenged at older ages. This was clearly shown in the severe lesions in H5N1-vaccinated groups challenged using the 2018 H5N8 isolate at two WPV compared to three WPV, where the lesions were between mild and moderate. This finding supported the risk of early infection in the gap before the appearance of high cross antibody levels [10]. In the case of the H5N2-vaccinated group, we found, as speculated, a relationship between the percentage of protection offered by the vaccine and its ability to reduce the pathogenicity of the virus in the internal organs of the birds challenged by the H5N8 2018 isolate (only 70% protection), particularly at 3 and 5 days PC, when the mortalities started as shown in Figure-1. Overall, the findings of our study have shown the matching between the severity of lesions and the shedding pattern.

While the inquiring results were the same lesions scored in H5N3-vaccinated groups at the same time with the presence of reasonable titers of the antibodies, which confirmed that the antibody level cannot be assumed to be the only factor indicating protection; either protection from lethal effects or minimizing of the virus spread from the portal of infection to the various different internal organs, which increases the severity of the disease [36,37]. HA is the major surface glycoprotein of AIV; it mediates both binding of the virion to host cell receptors and fusion between the virion envelope and endosomal membranes and, hence, is considered as being the most important protein in determining the virulence of AIVs [5]. The viral pathogenesis series is more notable in HPAIV infection, as it is well known that HA for influenza viruses is produced as a precursor, HA0, which requires cleavage by host proteases before it becomes functional and virus particles become infectious [33]. The fact which coincided with aforementioned studies has reported that circulating H5 and H7 LPAI viruses in gallinaceous poultry (chickens, turkeys, quail, etc.) might be able to mutate producing HPAIVs which can cause severe systemic disease and high levels of mortality in gallinaceous poultry and are typically easily transmissible [38]. Due to the ability of clade 2.3.4.4 viruses to mutate and reassort, various different genotypes and subtypes have emerged during their spread from Asia to Europe and Africa [39,40].

Although the two challenge isolates used in this study were isolated in Egypt at close points in time, this study triggered the necessity to assess the clear evolution of those newly emerging isolates and specifically of the antigenic sites that mainly govern their reaction to the same vaccines used in groups challenged with the H5N8 2018 isolate compared to those challenged with the 2017 isolate on the same age and also the same period from the vaccination time with taking in consideration that the field complications of the concurrent infection at the same flock [10,41]. Moreover, the inadequate biosecurity measures in the field are expected to reduce the performance of the vaccine used and insufficient laboratory evaluation of such vaccines [10].

Our findings have shown that vaccines exhibited reduced reactivity in most groups challenged with the new emerging viruses inferred as they can only survive the challenge. However, not at any time of the infection and not against all the virus isolates as showed with the most recent isolates reported in Egyptian HPAIV H5N8 2018 isolate which was used to evaluate the vaccine protective efficacy. In addition to immunogenicity and underlined the inefficacy of those vaccines to prevent or reduce the virus shedding to the limit that no vaccine-induced escape mutants detected anymore in all groups; excluding the groups not affected by the action of the vaccines on the side of lethal effect or the virus shedding on the other side. These issues have raised an urgent need for genetic characterization of the Egyptian HPAI H5N8 2018 isolates. There is a need to update the virus seeds used against the current AI infections and to customize attention to the new emerging H5N8 viruses.

## Conclusion

This study reports the differences in the efficacy of H5 vaccines against the 2018 isolates of HPAIV H5N8. The study also highlights the adaptive changes and pathogenicity of H5N8 in chickens. The increased pathogenicity of H5N8 strains is due mainly to the adaptation of these viruses in both vaccinated and non-vaccinated hosts. The use of inappropriate and ineffective H5 vaccines will contribute to such adaptation. There is a most imperative need to update and continuously evaluate the vaccines used to confer protection against HPAI H5N8 viruses.

## Authors’ Contributions

AAE: Conducted the experiment and drafted the manuscript., HAH and AHE: Designed and followed up the experiment and critically reviewed the manuscript. MFE, RAS, and MME: Participated in designing and followed up the practical work. This work is part of the master’s degree of AAE supervised by HAH, AHE, and MME. All authors read and approved the final manuscript.
